# Introduction to the Special Issue: The role of soil microbial-driven belowground processes in mediating exotic plant invasions

**DOI:** 10.1093/aobpla/plv052

**Published:** 2015-05-15

**Authors:** Inderjit

**Affiliations:** Department of Environmental Studies, Centre for Environmental Management of Degraded Ecosystems (CEMDE), University of Delhi, Delhi 110007, India

**Keywords:** Invasion, soil microbial communities, soil processes

## Abstract

Soil microbial communities are one of the multiple factors that facilitate or resist plant invasion. Regional and biogeographic studies help to determine how soil communities and the processes mediated by soil microbes are linked to other mechanisms of invasion. Both the success of plant invasions and their impacts are profoundly influenced by a wide range of soil communities and the soil processes mediated by them. With an aim to better understand the mechanisms responsible for the soil community-driven routes, a special issue of *AoB PLANTS* was conceived. I hope that the range of papers included in the special issue will reveal some of the complexities in soil community-mediated plant invasion.

## Introduction

Soil microbial communities play an important role in explaining why some exotic species become aggressive invaders and form mono-dominant communities in non-native ranges but coexist with their neighbours in species-diverse native communities. Research on invasion ecology during last two decades has made a significant contribution in understanding how soil communities can either resist or facilitate invasions (Bever 2003; Reinhart and Callaway 2006; Inderjit and van der Putten 2010; Bever *et al*. 2012; Van der Putten *et al*. 2013). Soil communities, mainly arbuscular mycorrhizal fungi (AMF), bacterial and fungal species, influence invasion by the presence or absence of virulent pathogens, culturing soil biota that exert positive or negative feedbacks, mutualists and the ability of soil microbial communities to influence nutrient availability and soil properties. In order to summarize recent advances in understanding the role of belowground processes in plant invasion, it is important to elucidate the mechanisms soil communities may employ to impact the establishment and colonization of invaders in non-native ranges. Biogeographic comparisons of ecological roles of soil communities in native vs. non-native ranges in combination with empirical studies testing their activities will help in understanding the soil communities as a driver of invasion in evolutionary contexts. Soil communities are largely coevolved in the native range of the exotic species, largely naïve in early phases of invasion and can become resistance in sites in non-native ranges with long invasion history (Inderjit and van der Putten 2010; Lankau 2011*a*). van der Putten (2012) identified three phases of the invasion of range-shifting species in non-native ranges. These are: introduction, boom and bust. The tens rule predicts that ≈10 % introductions are successful, and out of ≈10 % successful invasion ≈10 % become invasive (Williamson and Fitter 1996; Jarić and Cvijanović 2012). This suggests that ≈10 % of the species could overcome geographic barriers and arrive in a non-native range. Soil communities are one of the multiple factors that contribute to plant invasiveness. In the introduction phase, an invasive or range-shifting species has an advantage of enemy release (Mitchell and Power 2003; Reinhart *et al*. 2010), may have no biotic resistance, presence of generalist mutualists (Van der Putten 2012). Upon introduction to non-native ranges, an exotic species may gain advantage of escaping virulent pathogens present in its native range (Reinhart *et al*. 2010). An escape from virulent pathogens present in native range could help an exotic species to establish in the early phases of invasion. There is, however, a possibility that invader may encounter virulent pathogens in the non-native ranges, which does not allow exotic species to establish. This could be one of the causes why few exotics (≈10 % of the total ≈10 % successful introductions to non-native ranges) reproduce and could become invasive. Invasive species, however, may benefit from escaping enemies through all phases of the invasion process and not just the first phase. Likewise, some level of biotic resistance may also act on a plant on all phases of invasion but may not entirely restrict the invader unless the intensity of the negative effect increases or other negative effects such as herbivory gradually increase (K. Reinhart, pers. comm.).

In the boom phase, the positive benefits of introduction phase increase to facilitate invasive species by evolving increased competitive ability (Blossey and Nötzold 1995), invasive species experience a home field advantage due to specialization to decomposers and the presence of generalist mutualists (Van der Putten 2012). Once an exotic species becomes established and starts reproducing and expanding its range, an invader could culture soil biota that facilitate invasion (Reinhart *et al*. 2003; Callaway *et al*. 2004). Rare and endangered species may experience negative soil feedbacks compared with invasive species (Klironomos 2002). Some invaders accumulate native pathogens that suppress the establishment and growth of native seedlings (accumulation of native pathogens hypothesis; Eppinga *et al*. 2006; Mangla *et al*. 2008). The initial advantage of accumulating native pathogens in the early phases of invasion might turn into a disadvantage when the pathogens become specialized on their new hosts (Diez *et al*. 2010; Dostál *et al*. 2013).

Invaders such as garlic mustard (*Alliaria petiolata* (M. Bieb.) Cavara & Grande) suppress mycorrhizae and disrupt mutualistic associations between mycorrhizae and native tree seedlings (disruption of mutualistic associations; Stinson *et al*. 2006; Hale and Kalisz 2012). Some mutualists exert more positive impacts on the invader in the non-native ranges than their native ranges (enhanced mutualistic hypothesis; Reinhart and Callaway 2006; Sun and He 2010). Invaders can influence nutrient availability by releasing or depleting soil nutrients (Bajpai and Inderjit 2013; Mehrabi and Tuck 2015). In the early phase of invasion soil microbial communities therefore provide competitive advantage to the invader largely in ecological and evolutionary contexts as explained above (Van der Putten 2012).

In the bust phase range-shifting/invasive species may experience new enemies, which could be a major disadvantage because of their poor defense against enemies thus resulting in the decline in invasiveness (Van der Putten 2012). Sufficient evidence suggests a decline in invasiveness over a period of time (Simberloff and Gibbons 2004; Hawkes 2007; Lankau *et al*. 2009; Diez *et al*. 2010; Dostál *et al*. 2013). In the bust phase the impact of an invader might decline. Over a period of time, invasive species accumulate higher densities of pathogens and a higher likelihood of encountering pathogens that regulate its further spread (pathogen accumulation and invasive decline hypothesis; Flory and Clay 2013). An invader has a higher probability to cultivate pathogens in the presence of congeneric native species in non-native ranges (Parker and Gilbert 2007). Garlic mustard-invaded soils with a long invasion history (i.e. oldest invaded sites) showed recovery in richness of soil microbial communities (Lankau *et al*. 2009; Lankau 2011*b*). With longer invasion history a decline in the production of allelochemicals by garlic mustard was observed, which may reduce the impact of invader on soil microbial communities (Lankau *et al*. 2009; Lankau 2010). There is a need to study the differential response of different members of soil communities—AMF, bacteria and fungi—with longer invasion history. The invader establishes and undergoes range expansion and then finally contacts the rare or new pathogen in the non-native ranges. The pathogen may then gradually expand its range to match its new host leading to a decline in the invasiveness of exotic species. Invasive species eventually encounter rare pathogen genotypes thus supporting the evolutionary component of the decline in invasiveness (K. Reinhart, pers. comm.).

## Special Issue on the Role of Belowground Processes in Invasion

There is convincing evidence for the impact of soil communities on exotic plant invasion (Inderjit and van der Putten 2010; Bardgett *et al*. 2013; Brandt *et al*. 2013; Fukami and Nakajima 2013; Hendriks *et al*. 2013; Kardol *et al*. 2013; Suding *et al*. 2013; Van der Putten *et al*. 2013). Some soil community-driven mechanisms of invasion such as impact on soil fertility, tri-trophic interactions and identification of soil pathogens needs more attention to unravel linkages between soil communities-driven mechanisms and other underlying mechanisms of plant invasion. Plant–soil feedbacks, negative or positive, are generally studied by comparing growth in non-sterile vs. sterile soil from non-native and/or native ranges, and by taking home vs. away approaches (Johnson 2010; Callaway *et al*. 2011). The qualitative and quantitative data on soil biota are not always available. Kardol *et al*. (2013) discussed the significance of identification of soil organisms involved in PSFs. These authors suggested that inoculation experiments with identified soil organisms should be designed to vary plant and soil microbes and responses should be monitored over a period of time. It is also important to further understand how soil communities drive nutrient fluctuations mainly nitrogen, responsible for positive or negative feedbacks (Bajpai and Inderjit 2013; Mehrabi and Tuck 2015).

To provide a current overview on the ecological role of belowground processes, largely driven by soil communities and to overcome lacuna/difficulties in carrying out research on the influence of soil communities on invaders, a special issue was conceived. Papers were invited on some relevant ecological areas of soil communities and invasion. The invasional meltdown hypothesis predicts that one invader facilitates the invasion of another. **Dickie, St John, Yeates, Morse, Bonner, Orwin and Peltzer** discuss the belowground legacies of an invader *Pinus contorata* driven by co-invasion with mycorrhizal fungi and changes in soil chemistry. In an elegant study, these authors found that invader *P. contorata* promotes ectomycorrhizal infection of another invader *P. menziesii* but neither of conspecific *Pinus* species nor of native *Kunzea ericoides*. However, the authors also found a second feedback mechanism involving soil nutrients, with invasive *Pinus* enhancing the levels of nitrate-nitrogen and available phosphate-phosphorus. This belowground legacy of *P. contorta* appears to be driving increased invasion by non-native grasses following tree removal. This study nicely illustrates the possible soil community-driven invasional meltdown mechanisms.

In some situations, an exotic invasive can suppress another exotic invasive. **Blank, Morgan and Allen** studied the causes of the suppression of exotic annual *Bromus tectorum*, which is responsible for fire in the rangeland in western USA. *Bromus tectorum* cannot easily establish in lands dominated by the exotic perennial grass *Agropyron cristatum*. These authors found that *A. cristatum* suppresses *B. tectorum* by lowering soil nitrate-nitrogen and nitrite-nitrogen availability. **Dickens, Allen, Santiago and Crowley** showed that the removal of invasives in grasslands leaves legacy effects by changing soil microbial and nitrogen cycling characteristics that make soils prone to invasion by exotic annuals.

Activated carbon is often employed in allelopathy research to invoke the role of allelochemicals in plant growth suppression (Inderjit and Callaway 2003; Lau *et al*. 2008; Wurst *et al*. 2010). **Nolan, Kulmatiski, Beard and Norton** found that activated carbon decreased non-native plant growth in both field and laboratory settings, but that this effect appeared to occur only in live, not sterile, soils. This suggested that for the 10 common native and non-native plant species studied, plant–microbe interactions were critical to observed plant responses. These authors carried out genetic analyses and suggested that an unidentified bacterium, an Actinomycetales and a *Flavobacterium* were likely to play a role in this plant response to activated carbon treatment. Results suggest that activated carbon may have a larger effect on plant–microbe interactions than on allelopathy alone.

One of the hypotheses that links novel chemicals and suppression of root-fungal symbioses is disruption of mutualistic hypothesis (Stinson *et al*. 2006). **Brouwer, Hale and Kalisz** in a greenhouse experiment found that allelochemicals released by garlic mustard can create carbon stress in terms of total non-structural carbohydrates (inulin and sucrose) by disrupting mutualistic root-fungal symbioses in native plant species. Similar results were obtained in a long-term field experiment: where garlic mustard was present, vital rates of native perennials were suppressed relative to weeded plots, suggesting a general link between the chemically driven disruption of the mutualism and plant physiological declines, carbon stress and demographic declines.

The impact of soil communities on invasion is linked to other mechanisms of plant invasions. **Inderjit and Cahill** gave an overview of plant–soil feedbacks linkages to invasion mechanisms. The authors stressed that PSF impacts need to be evaluated in the context of herbivores, competitors, chemicals, pollinators and soil heterogeneity in temporal and spatial scales. In an interesting study on how any change in soil microbial communities along soil nitrogen gradient would influence PSF, **Larios and Suding** concluded that plant species hosting soil communities and soil resources may impact soil microbial communities, and neighbouring competing species also influences such effects. Studies have shown increasing negative plant–soil feedback to occur when invaders have spent increasing amounts of years in their new range. Moreover, Klironomos (2002) showed that abundant species have less negative feedback than rare species (but see Reinhart *et al*. 2012). In the study by **Speek, Schaminée, Stam, Lotz, Ozinga and van der Putten** detailed information on time since introduction and local abundance in the Netherlands was provided. These authors, however, could not confirm whether, how and when time since local dominance of exotic plant species may relate to plant–soil feedback.

**Vestergård, Rønn and Ekelund** separated invasion into irreversible and reversible processes. In the case of irreversible invasion, decline of native species is largely irreversible. When the impact of invasion starts declining over a period of time, invasions are identified as reversible. These authors highlighted the importance of studying invasion in an ecological and evolutionary context.

I hope that this special issue of the *AoB PLANTS* provides some novel insights into current research on soil microbial-driven belowground processes mediating plant invasions and reveals areas that need attention in future studies.

## Sources of Funding

The Council of Scientific & Industrial Research (CSIR) and the University of Delhi supported this work.

## Conflict of Interest Statement

None declared.
