# Corrigendum

**DOI:** 10.1093/aobpla/plv093

**Published:** 2015-08-21

**Citation**: Inderjit. 2015. Introduction to the Special Issue: The role of soil microbial-driven belowground processes in mediating exotic plant invasions. *AoB PLANTS* **7**: plv052; doi:10.1093/aobpla/plv052

One of the articles in the special issue (plv062: Putative linkages between below and aboveground mutualisms during alien plant invasions, by Susana Rodríguez Echeverría and Anna Traveset) was not included in the previous version of the introduction. A revised version which includes the missing article has been published, and we apologize for the omission.

